# Postpneumonectomy-like syndrome due to bronchial carcinoid: a unique case report

**DOI:** 10.1186/s12890-018-0767-5

**Published:** 2019-02-18

**Authors:** Athanasios K. Konstantinidis, Vlasios V. Vitsas, Konstantinos Tatsis, Thomas Vadivoulis, Apostolos Kittas, Christos Chronis, Vanesa Bellou, Ioannis N. Vamvakaris, Rodoula Tringidou, Grigoris K. Stratakos

**Affiliations:** 1 Division of Pulmonary Medicine, University of Ioannina School of Medicine, University Hospital of Ioannina, S. Niarhou Ave, 45500 Ioannina, Greece; 2 1st Respiratory Medicine Department, University of Athens, “Sotiria” Hospital, 152 Mesogeion Ave, 115 27 Athens, Greece; 3 Department of Radiology, University Hospital of Ioannina, S. Niarhou Ave, 45500 Ioannina, Greece; 4 Department of Pathology, “Sotiria” Hospital, 152 Mesogeion Ave, 115 27 Athens, Greece

**Keywords:** Postpneumonectomy-like syndrome, Postpneumonectomy syndrome, Bronchial carcinoid

## Abstract

**Background:**

Postpneumonectomy-like syndrome is a rare condition resulting from unilateral lung disease with severe lung volume loss leading to excessive mediastinal shift and herniation of the healthy lung into the contralateral hemithorax, mimicking the mediastinal shift observed in postpneumonectomy syndrome after pneumonectomy. We report a unique case of postpneumonectomy-like syndrome caused by an atypical bronchial carcinoid completely occluding the left main bronchus.

**Case presentation:**

A 25-year-old woman presented with symptoms of chronic exertional dyspnea and productive cough. Imaging studies showed complete left lung atelectasis due to a mass occluding the left main bronchus, as well as extreme mediastinal deviation and substantial herniation of the right lung into the left hemithorax. Bronchoscopic biopsy of the tumor and subsequent left pneumonectomy with concurrent lymph node dissection revealed an atypical carcinoid. Sixteen months after surgery the patient has been asymptomatic with repeat imaging studies showing no change in mediastinal shifting.

**Conclusion:**

Bronchial carcinoids are notorious for causing bronchial obstruction. The present case represents an extreme complication of centrally located bronchial carcinoid, resulting in postpneumonectomy-like syndrome with severe mediastinal shift and herniation of the healthy lung into the diseased hemithorax.

## Background

Lung carcinoids are considered rare tumors with an annual incidence comprising between 2.3 and 2.8 cases per 1 million people; they include 20 to 25% of all carcinoid tumors throughout the body but account to only 0.4 to 3% of all primary lung cancers [1]. Typical carcinoids account for the majority of carcinoid tumors and are approximately four to eight times more frequent than atypical carcinoids [2]. Lung carcinoids are the most common primary lung neoplasm in children and late adolescents, with typical carcinoids prevailing by far over atypical carcinoids [3]. Carcinoid syndrome is found in 2–5% of pulmonary carcinoids, most often when liver metastases are present. Pulmonary carcinoids may be rarely associated with MEN1 syndrome (1–5% of patients), while Cushing’s syndrome is found in 1–6% of patients [3]. Approximately 75% of patients with carcinoid tumors present with central tumors and symptoms of cough, hemoptysis, wheeze, recurrent pneumonia or chest pain [2]. Radiologic findings are usually related to bronchial obstruction and include associated atelectasis, air trapping, obstructing pneumonia and mucoid impaction [4]. Postpneumonectomy-like syndrome is a rare condition caused by either destructive or neoplastic unilateral lung disease or by congenital lung agenesis or hypoplasia, resulting in herniation of the normal lung into the diseased hemithorax with compensatory hyperinflation and air trapping in that lung [5]. Its pathogenic mechanism and treatment are analogous to those of postpneumonectomy syndrome, but without previous lung resection [6]. Here, we describe a unique case of postpneumonectomy-like syndrome characterized by complete left lung collapse, severe mediastinal deviation and significant lung herniation into the left hemithorax due to atypical carcinoid in the left main bronchus in a 25-year-old woman.

## Case presentation

A 25-year-old female presented to the emergency department (ED) for evaluation of persistent productive cough of yellowish sputum over the last four week and mild exertional dyspnea over the last two years. Her past medical history was unremarkable and she took no regular medications. There was no personal or family history of multiple endocrine neoplasia type 1 (MEN1) syndrome. She was in no distress on presentation to the ED with a resting hemoglobin oxygen saturation of 97% while breathing room air. Her physical examination was remarkable for absent breath sounds and decreased tactile fremitus on the left middle and lower lung fields. No wheezing or stridor were heard. Laboratory data were within normal limits.

**Fig. 1:**
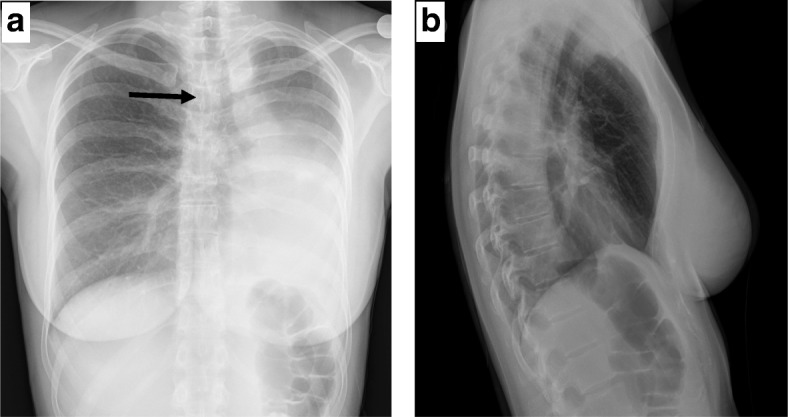
**a**. Chest radiograph (postero-anterior view) showing opacification of the left middle and lower lung zone. Tracheal deviation towards the left is also evident; (black arrow). **b**. Lateral chest radiograph

**Fig. 2:**
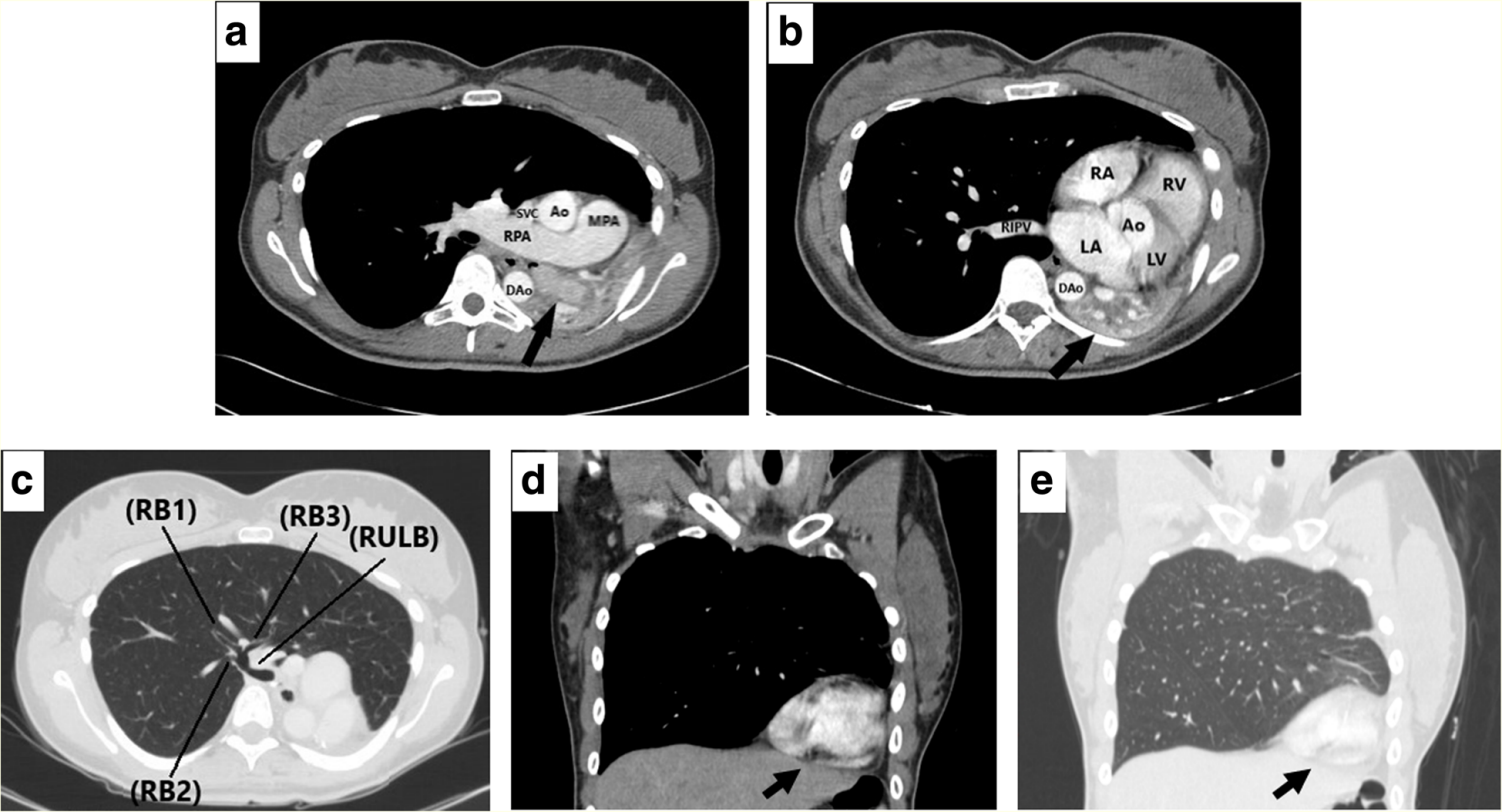
**a**. Axial contrast-enhanced chest CT scan with mediastinal-windowing shows an enhanced endoluminal mass within the left main bronchus (black arrow) and clockwise mediastinal shift. MPA, main pulmonary artery; RPA, right pulmonary artery; Ao, ascending aorta; DAo, descending aorta; RIPV, right inferior pulmonary vein. **b**. Axial chest CT scan with mediastinal-windowing demonstrates the completely collapsed left lung lying posterior to the heart (black arrow). Ao, ascending aorta; RA, right atrium; RV, right ventricle; LA, left atrium; LV, left ventricle; DAo, descending aorta. **c**. Axial chest CT scan section at the level of the right upper lobe imaged with lung window shows clockwise mediastinal shift with herniation of the right upper lobe into the left hemithorax. RULB, right upper lobe bronchus; (RB1), right upper lobe apical segmental bronchus; (RB2), right upper lobe posterior segmental bronchus; (RB3), right upper lobe anterior segmental bronchus. **d**. Coronal contrast-enhanced chest CT scan with mediastinal-windowing, shows extreme mediastinal shift towards the left and significant clockwise rotation of the heart (black arrow). **e.** Coronal contrast-enhanced chest CT scan imaged with lung window, shows overexpansion and substantial herniation of the right lung into the left hemithorax as well as severe clockwise rotation of the heart (black arrow)

**Fig. 3:**
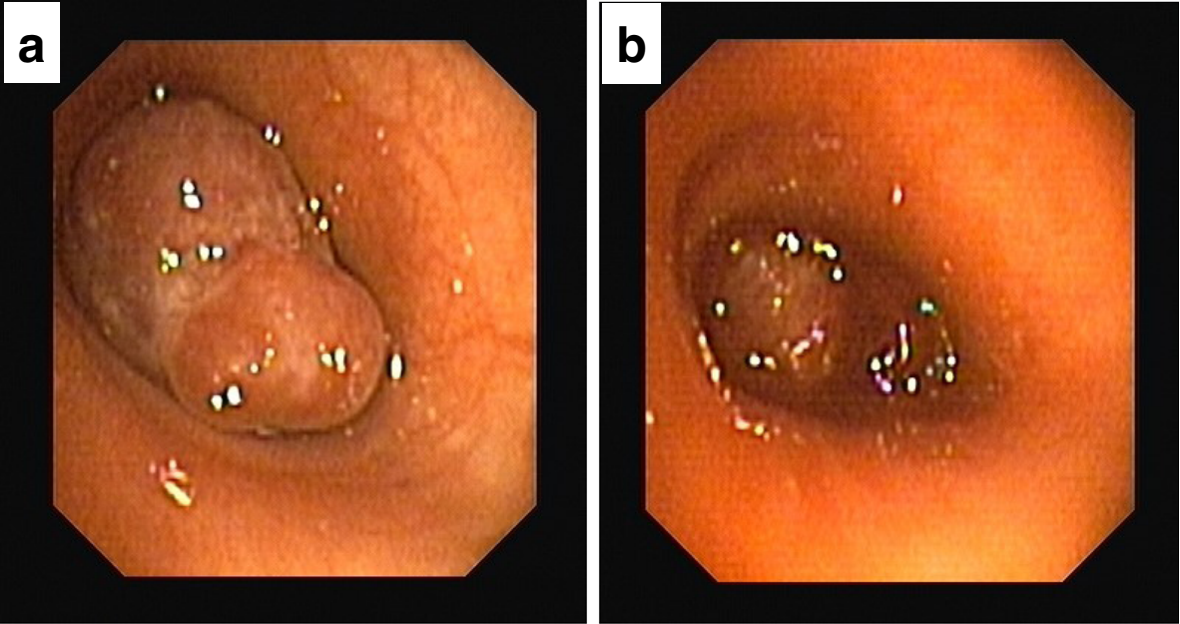
**a**. Bronchoscopic image shows a polypoid tumor completely occluding the left main bronchus. **b**. Bronchoscopic image of the same tumor right after forceps biopsy

A chest x-ray (CXR) in the ED demonstrated opacification of the left middle and lower lung fields, hyperinflation of the right lung and deviation of the trachea to the left (Fig. 1). A computerized tomography (CT) scan of the chest showed complete left lung atelectasis due to a mass obstructing the left main bronchus and excessive mediastinal deviation to the left with substantial herniation of the hyperdistended right lung into the left hemithorax (Fig. 2). There was no evidence of tracheobronchial narrowing in the right lung or esophageal compression. The mass was well demarcated and of soft-tissue quality, demonstrating homogeneous contrast enhancement, starting 2.8 cm distal to the main carina, measuring 4.4 × 2 × 2.8 cm (Fig. 2). Abdominal and head CT scans showed no abnormal findings. The patient subsequently underwent a diagnostic flexible bronchoscopy which revealed a pale hypervascular polypoid mass completely obliterating the left main bronchus which was biopsied using forceps (Fig. 3). Histopathological examination of endobronchial biopsies disclosed a carcinoid tumor with a Ki-67 index of approximately 10%.

**Fig. 4:**
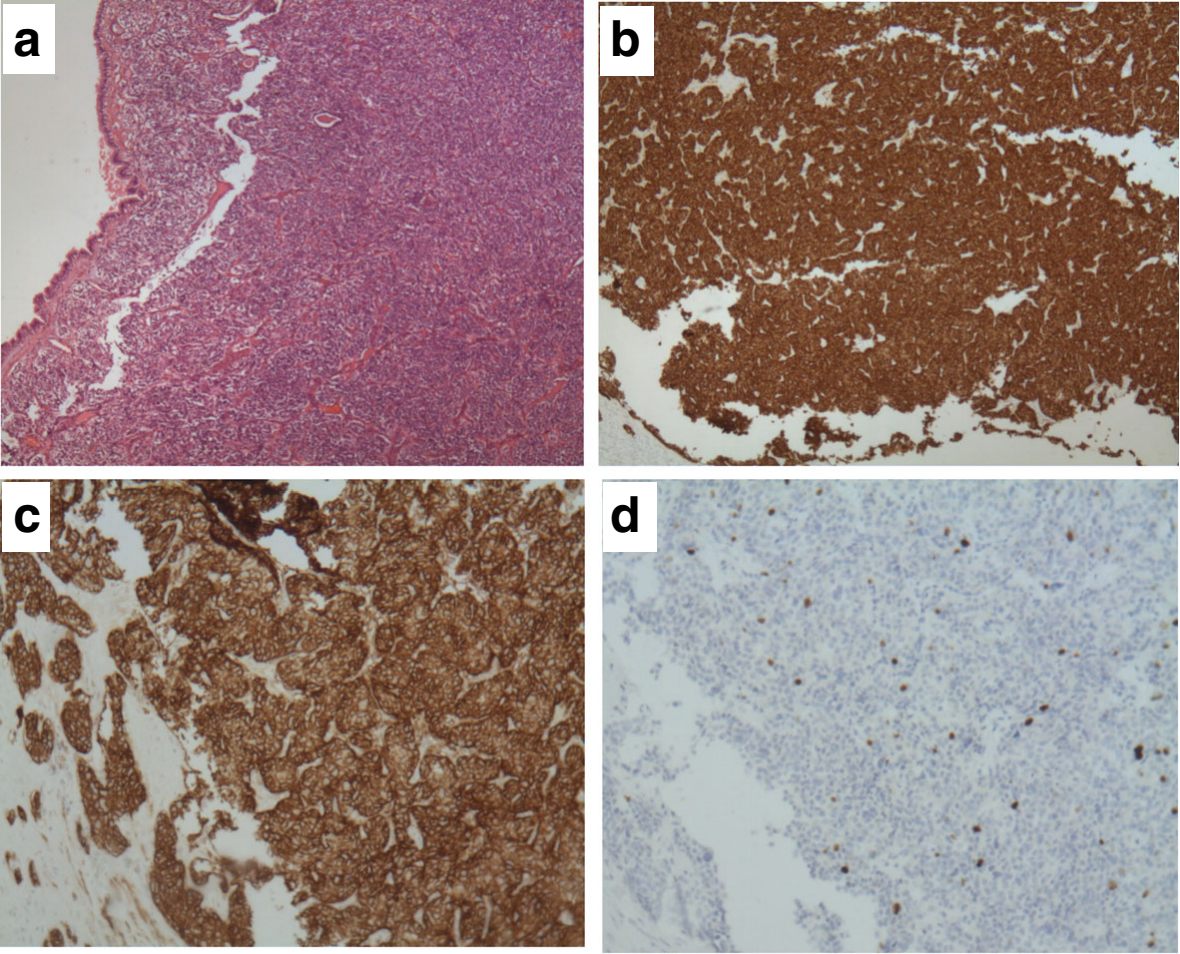
Histology of bronchial atypical carcinoid tumor. **a**. Small rounded uniform cells and highly vascularized stroma are seen. Bronchial epithelium on the left appears intact. (hematoxylin-eosin**)**. **b**. Strong synaptophysin expression in tumor cells. **c**. Bright CD56 expression in tumor cells. **d**. Ki67 immunostaining in tumor cells was approximately 10% (original magnifications × 40 [A through D])

Following thoracic surgery consultation, an open left pneumonectomy with concurrent complete lymph node assessment and dissection was performed. During surgery, the left lung was found completely atelectatic with adhesions between the pericardium and the left pleura which were dissected. No attempt of repositioning the mediastinum or placement of tissue expanders was performed, due to the absence of airway compression in the right bronchial tree during previous bronchoscopy and CT scan. The patient recovered well after surgery and no complications were noted. Post-operative histopathology disclosed an atypical carcinoid with a Ki-67 labelling index of 10% but no areas of necrosis (Fig. 4). There was a radical resection of all tumor with clear operative margins, the periphery of the left main bronchus was infiltrated by tumor, but there was no invasion of the visceral pleura, and no infiltration of resected lymph nodes from lymph node stations 5, 7, 9 and 10 by carcinoid cells.

Pre-operative spirometry was as follows: FEV_1_: 1.51 lit (44% predicted), FVC: 1.54 lit (39% predicted), FEV_1_/FVC: 98%. Spirometry and static lung volumes 12 months after surgery were as follows: FEV_1_: 1.93 lit (58% predicted), FVC: 2.34 lit  (61% predicted), FEV_1_/FVC: 82%, TLC: 3.28 lit (63% predicted), RV/TLC: 118% predicted. Although spirometry appears to be significantly improved after surgery, spirometry before surgery triggered fits of coughing and therefore preoperative values might not be representative.

Postsurgical follow-up has included the following: Initial chest CT scan was carried out 2 months after surgery. Parathyroid hormone (PTH) and prolactin levels were within normal limits 1 year after surgery. The following investigations were carried out at 6 months and then every 6 months for the first 5 years: Chest CT scan, abdominal ultrasound, chromogranin A measurement and standard laboratory testing including complete blood count, renal function, liver function, calcium and glucose. Abdominal CT scan and fiberoptic bronchoscopy were carried out 1 year after surgery and then will be carried out annually for the first 5 years. Bronchoscopy would be performed earlier for any symptoms or imaging findings suggestive of local progression. Repeat chest CT scans after surgery showed no changes in mediastinal rotation compared to those prior to surgery, and no signs of tracheobronchial or esophageal compression. Repeat bronchoscopy showed a normal-appearing surgical stump of left main bronchus and no airway compression of the right bronchial tree. The remaining studies listed above have been normal. The chronic mild exertional dyspnea reported by the patient before surgery completely resolved on hospital discharge, 7 days after pneumonectomy. The patient has been asymptomatic for the last 16 months after surgery with excellent performance status.

## Discussion

According to the last WHO 2015 classification of tumors of the lung, pleura, thymus and heart [7], neuroendocrine tumors of the lung are neuroendocrine epithelial malignancies and are separated into four major categories: small-cell lung carcinoma (SCLC) and large-cell neuroendocrine carcinoma (LCNEC) (which are high-grade neuroendocrine tumors) and typical carcinoid tumor (TC) and atypical carcinoid tumor (AC) (which are considered to be low- and indermediate-grade malignant tumors, respectively). Neuroendocrine differentiation of carcinoids is established by immunohistochemical identification of secreted and cytoplasmic products such as synaptophysin, neuron-specific enolase, and chromogranin [8]. Carcinoid tumors are divided into two subcategories: typical carcinoid tumors with < 2 mitoses per mm^2^ and lacking necrosis, and atypical carcinoid tumors with 2–10 mitoses per mm^2^ and/or foci of necrosis. The Ki-67 antigen identifies proliferating cells spanning from G1 to M phase and is valuable in biopsy samples with crush artifact, where mitotic index is difficult to assess, and may play a role in predicting prognosis [3]. The recent WHO 2015 classification mentions that a tumor with carcinoid-like morphology has a low labelling index (< 10–20%). However, although Ki-67 labelling index cut-off values ranging from 2.5 to 5.8% have been proposed in the literature, the utility of this marker to discriminate typical carcinoid from atypical carcinoid or to predict prognosis is not established [7, 9]. However, at the individual patient level, none of these features enables a reliable prediction of clinical outcome as tumor recurrence was found to correlate significantly not only with the carcinoid histotype and mitotic index, but also with tumor location, necrosis, tumor vascular invasion and synchronous mediastinal nodal metastasis [10]. Typical carcinoids rarely metastasize and exhibit a 5-year survival rate ranging between 97 and 100%, whereas patients with atypical carcinoids have a greater tendency to present with lymph nodal involvement and distant metastases, usually to liver and bone, and have a 5-year survival rate varying from 25 to 70% [1].

In our patient, we proceeded with surgery rather than bronchoscopic resection of the tumor (EBT), for two reasons. Firstly, chest CT scan showed a purely intraluminal growth of the tumor, which was confirmed by final pathology, with the mass starting 2.8 cm distal to the main carina. However, tumor size was large enough, measuring 4.4 × 2 × 2.8 cm, and infiltrated the periphery of the main bronchus on chest CT scan and on final pathology. Secondly, biopsies taken during initial bronchoscopy showed a high Ki67 index, suggestive of an atypical carcinoid. We thus decided that the patient was not eligible for EBT, as this modality may have left substantial tumor behind. Our preoperative plan is supported by the findings of a large cohort of one hundred and twenty-five patients with a diagnosis of bronchial carcinoid who underwent endobronchial treatment [11]. In this cohort, no patient with a tumor diameter ≥ 20 mm was successfully treated with EBT, and the authors of that study recommended that these patients should be directly referred for surgery [11].

Given the young age and the rather limited pre-surgical pulmonary function of our patient [pre-surgical FEV_1_: 1.51 lit (44% pred.)], a parenchymal-sparing surgery, such as left main bronchus resection and reconstruction, along with nodal dissection might have been the ideal surgical treatment. However, a left pneumonectomy rather than a parenchymal sparing surgery was performed because chest CT scan showed infiltration of the periphery of the left main bronchus by tumor, which would not have allowed reconstruction of the left main bronchus and reareation of the atelectatic lung because there was no free margin adequacy. Infiltration of distal main bronchus was subsequently confirmed by final pathology.

Postpneumonectomy syndrome is a rare complication of pneumonectomy. Rotation of the heart and great vessels and herniation of the remaining lung into the contralateral hemithorax may produce compression of the distal trachea and main stem bronchus between the vertebral column and the aorta posteriorly and the pulmonary artery anteriorly, causing symptomatic airway stenosis or less frequently esophageal compression [12, 13]. Symptoms include progressive shortness of breath, stridor, dysphagia and syncope, but some patients remain asymptomatic [12–14]. This syndrome also occurs in the absence of pneumonectomy, due to unilateral destructive or neoplastic lung disease or due to congenital lung agenesis or hypoplasia, in which case it is termed postpneumonectomy-like syndrome [6]. We conducted a search of the PubMed database using the term: “postpneumonectomy-like syndrome” and retrieved a total of seven cases, not related to congenital lung agenesis or congenital lung hypoplasia. In four cases the mediastinum was shifted to the right and in three cases to the left. One patient with destructed lung due to tuberculosis died 4 days after admission and five patients required surgical repositioning of the mediastinum. Four of those cases involved patients with unilateral lung destruction due to previously treated severe parenchymal tuberculosis [15–17]. Two cases involved patients with malignancy, one with long-standing history of Hodgkin’s lymphoma involving the left lung and mediastinum [18] and one with lung cancer [6]. Another case involved a patient with destruction of the right lung due to multiple episodes of pneumonia [19] Several cases of postpneumonectomy-like syndrome due to congenital lung agenesis or hypoplasia have been described [5, 20, 21]. Among seventy-three cases of postpneumonectomy syndrome in adults reported in the literature, the median interval between pneumonectomy and onset of symptoms was 2 years (range 1 month—49 years) [14]. Among the seven cases of postpneumonectomy-like syndrome reported in the literature, the time between start of the underlying disease and the onset of postpneumonectomy-like syndrome ranged from two months to thirty years. The longest time interval involved patients with long-standing destructive pulmonary tuberculosis, however, in those cases the authors have not provided either initial or sequential imaging studies, therefore, it is not known if those patients had gradual volume loss in the affected lung over time or not. The time between start of carcinoid and the diagnosis of the syndrome in our patient is unknown, since she had not previously undergone chest imaging studies.

Static lung volumes and capacities 12 months after surgery were higher than expected reflecting overexpansion of the right lung shown in chest imaging studies. The young age and female sex of our patient have played a key role in the development of the syndrome; given that postpneumonectomy syndrome following pneumonectomy occurs more frequently in infants, young children, and women due to the increased elasticity and compliance of their lungs and mediastinum compared with those of older patients and men [22]. The mild chronic exertional dyspnea on initial presentation completely resolved 7 days after pneumonectomy, indicating that it was most probably due to the shunt produced by the completely collapsed left lung.

The absence of symptoms and the lack of signs of tracheobronchial compression on imaging and bronchoscopy in our patient so far, despite the excessive mediastinal shift and the substantial herniation of the right lung into the left hemithorax, is not surprising. Firstly, the fact that the obstructed lung was the left one, explains the subtle symptoms of the patient as it is well reported that leftward shift of the mediastinum produces smaller anatomic and physiologic changes as the translocation of the heart and major vessels is smaller [12]. Secondly, these changes in our patient developed gradually overtime in a young and otherwise healthy individual thus giving her the time to smoothly adapt to the new situation. Previously reported cases of postpneumonectomy syndrome after pneumonectomy demonstrated a wide clinical spectrum ranging from absence of symptoms, as in our patient, to rapidly progressive shortness of breath [13]. In addition, not all patients with extreme mediastinal shifts observed in large series of pneumonectomies for cancer in adults require symptomatic treatment [23]. Although we considered implantation of prosthesis in the left mediastinum to maintain mediastinal position after pneumonectomy, we have not proceeded to surgical correction since the patient has remained asymptomatic with no evidence of bronchial or esophageal compression or stretching on repeat chest CT scans and bronchoscopy. However, mediastinal repositioning remains a valid surgical option if the patient develops symptoms or signs of bronchial or esophageal compression and stretching in the future.

To our knowledge, this is the first report of a bronchial carcinoid causing postpneumonectomy-like syndrome.

## Conclusions

Although bronchial carcinoids frequently cause bronchial obstruction, development of postpneumonectomy-like syndrome due to central bronchial obstruction, as in our patient, is an exceptional complication. Similar to postpneumonectomy syndrome following pneumonectomy, patients with postpneumonectomy-like syndrome exhibit a wide range of symptoms, from no complaints to severe shortness of breath or dysphagia requiring surgical repositioning of the mediastinum.
